# Geostatistical modelling enables efficient safety assessment for mass drug administration with ivermectin in *Loa loa* endemic areas through a combined antibody and LoaScope testing strategy for elimination of onchocerciasis

**DOI:** 10.1371/journal.pntd.0010189

**Published:** 2022-02-09

**Authors:** Olatunji Johnson, Emanuele Giorgi, Claudio Fronterrè, Benjamin Amoah, Julienne Atsame, Sylvie Ntsame Ella, Marco Biamonte, Kisito Ogoussan, Lee Hundley, Katherine Gass, Peter J. Diggle

**Affiliations:** 1 CHICAS, Lancaster Medical School, Lancaster University, Lancaster, United Kingdom; 2 Department of Mathematics, University of Manchester, Manchester, United Kingdom; 3 Control Program of Parasitic Diseases, Libreville, Gabon; 4 Drugs & Diagnostics for Tropical Diseases, San Diego, California, United States of America; 5 FHI 360, Washington, District of Columbia, United States of America; 6 Task Force for Global Health, Decatur, Georgia, United States of America; NIPD: National Institute of Parasitic Diseases, CHINA

## Abstract

The elimination of onchocerciasis through community-based Mass Drug Administration (MDA) of ivermectin (Mectizan) is hampered by co-endemicity of *Loa loa*, as individuals who are highly co-infected with *Loa loa* parasites can suffer serious and occasionally fatal neurological reactions from the drug. The test-and-not-treat strategy of testing all individuals participating in MDA has some operational constraints including the cost and limited availability of LoaScope diagnostic tools. As a result, a *Loa loa* Antibody (Ab) Rapid Test was developed to offer a complementary way of determining the prevalence of loiasis. We develop a joint geostatistical modelling framework for the analysis of Ab and Loascope data to delineate whether an area is safe for MDA. Our results support the use of a two-stage strategy, in which Ab testing is used to identify areas that, with acceptably high probability, are safe or unsafe for MDA, followed by Loascope testing in areas whose safety status is uncertain. This work therefore contributes to the global effort towards the elimination of onchocerciasis as a public health problem by potentially reducing the time and cost required to establish whether an area is safe for MDA.

## Author summary

Considering the serious adverse events that occur in individuals with high intensity of Loa loa parasite when being treated with ivermectin during the MDA program for the elimination of onchocerciasis, there is need for a comprehensive, safe and cost-effective strategy to delineate village or communities that are safe for MDA. In this study, we propose a hybrid strategy that uses information from the Loa antibody rapid test and the LoaScope diagnostic test to delineate whether an area is safe for MDA. We developed a joint geostatistical modelling framework that exploits both the association between antibody and LoaScope responses at community-level and the spatial correlation of the Loa loa prevalence surface to determine if the risk of observing individuals with high-intensity infections in a village or community is sufficiently low. Our results support the use of a two-stage strategy in which antibody test is used first as a screening tool and only those communities for which safety are in doubt are followed up with confirmatory LoaScope testing.

## Introduction

Loiasis is a major public health issue because of its geographic overlap with onchocerciasis and lymphatic filariasis [1]. The elimination of onchocerciasis through community-based Mass Drug Administration (MDA) of ivermectin (Mectizan) is hampered by co-endemicity of *Loa loa*, as individuals who are highly co-infected with *Loa loa* parasites can suffer serious and occasionally fatal neurological reactions from the drug.

Severe adverse events (SAEs) are largely confined to individuals whose *Loa loa* microfilaremia load exceeds 30,000 mf/mL as estimated using thick film blood microscopy [2]. A lower safety threshold has since been suggested whereby ivermectin should be given only to individuals with *Loa loa* microfilaremia loads less than 20,000 mf/mL [3]. Historically, the benefits of ivermectin treatment in reducing onchocerciasis-related blindness were deemed to out-weigh the risk of severe adverse events (SAEs) only in areas where onchocerciasis is hyper- or meso-endemic [1]. Hence, where Loa loa is co-endemic, the treatment strategy for onchocerciasis has been confined to areas of high prevalence, leaving a gap in the guidance for how to proceed where onchocerciasis is hypo-endemic.

Until recently, this test-and-not-treat (TaNT) strategy was infeasible at the required scale because of the need for trained microscopists to be available at the point of care. As a result, guidelines for the safe roll-out of MDA were based on estimated community-level prevalence using a low-cost questionnaire instrument, RAPLOA [4], which exploits the association between community-level *Loa loa* prevalence and the probability that an individual in that community will be highly infected. Schluter *et al* [5] developed a statistical model for the joint variation in community-level prevalence and the distribution of microfilaremia (Mf) loads among infected individuals in the community and showed that the model could be used to predict, with quantifiable uncertainty, the proportion of highly infected individuals in a community using only data on the presence/absence of Mf infection, albeit with lower precision than if individual-level data are available on both presence/absence and Mf load. Giorgi *et al* [6] extended the Schluter *et al* [5] approach by allowing the random effects in the model to be spatially correlated and showed that this improves prediction because data from one location are partially predictive of infection levels at nearby locations.

The invention of the Loascope, a mobile telephone-based field-friendly device that measures microfilarial counts [7] rendered a test-and-not-treat (TaNT) strategy feasible, whereby individuals are first tested by the Loascope and only those with mf counts below the safety threshold receive ivermectin. This TaNT strategy has been successfully tested in the field in Cameroon [3]. Nevertheless, shifting from a mass treatment strategy to an individual-based TaNT approach has major cost and resource implications for programs, making it impractical to implement more broadly.

A response to these challenges is to devise a hybrid strategy whereby communities are initially screened to estimate their *Loa loa* prevalence, and only those communities whose safety for MDA is in doubt are followed up using TaNT. A hybrid strategy has been rendered more attractive by the development of a new immunological indicator for current or past exposure to the Loa loa parasite, the *Loa loa* Antibody (Ab) Rapid Test developed by Drugs and Diagnostics for Tropical Diseases, San Diego, CA [8].

In this paper we propose a hybrid strategy that uses information from the Ab test and the LoaScope to delineate whether an area is safe for MDA. We demonstrate that by exploiting both the association between Ab and Loascope responses at community-level and the spatial correlation of the *Loa loa* prevalence surface leads to more precise prediction than can be made from either of the two data sources alone.

Fundamental to our approach is that prediction is always subject to a degree of uncertainty. A balance therefore needs to be struck between the risk to an individual of applying MDA incautiously and the risk to a community of withholding MDA unnecessarily. We therefore adopt the following definition: a community is *safe for MDA* if there is a probability at least *q* that a proportion at most *p* of individuals in the community are carrying at least *c* *Loa loa* microfilariae per ml of blood. In resource-limited settings, the use of efficient geostatistical methods can minimise the mis-classification of communities as safe or unsafe according to this definition, but wider economic, social and ethical considerations are needed to determine appropriate values for *q*, *p* and *c*. An emerging consensus from the Mectizan Expert Committee meeting held in Atlanta, Georgia, 27–29 April 2016, is that acceptable values for operational decisions are *q* = 0.95, *p* = 0.01 and *c* = 20, 000. A remaining consideration is to decide what constitutes a community. Practical considerations again require a balance to be struck, in this case between local and global decisions. The concept of an evaluation unit (EU) is well-established in MDA programmes, and can be variously defined as a set of adjoining districts, a single district or a sub-district.

Our objectives in the remainder of this paper are:

1. to develop a joint geostatistical modelling framework for the analysis of Ab (presence/absence) and Loascope (estimated mf load) data;
2. to show, using data from Gabon, how the model can be used to classify communities as *safe*, *unsafe* or *don’t know* for MDA using:
  1. Ab and Loascope data in combination;
  2. Ab data alone;
  3. a two-stage strategy in which Ab is used as a screening tool and only those communities for which safety is classified as *don’t know* are followed up with confirmatory Loascope testing.

## Methods

### Ethics statement

Ethical approval for the study was obtained from the institutional review committee affiliated with the Ministry of Health of Gabon [approval 0254].

### *Loa loa* diagnostics

The LoaScope is a smartphone-based microscope technology developed at University of California Berkeley. It uses video from a smartphone-connected microscope to automatically detect and quantify *Loa loa* microfilariae in peripheral blood [7]. It has an optional smartphone-based reader that allows users to capture GPS coordinates, time stamp, and transfer patient information to a secure server. At the time of writing, the Loascope has not received WHO formal approval for individual case management as it is primarily devised for epidemiological studies and to support mapping projects for ivermectin-based MDA programs.

The *Loa loa* Antibody (Ab) rapid test was developed by Drugs and Diagnostics for Tropical Diseases, San Diego, CA [8]. It has the advantage over microscopy-based diagnostics for *Loa loa* prevalence that it can be used at any time of day. However, as an antibody-based test it does not discriminate previous from current infections, and therefore estimates a higher level of prevalence than is estimated by the Loascope.

The sensitivity and specificity of the Loascope and of the Loa Ab rapid test have been examined by D’Ambrosio et al [7] and Pedram et al [8], respectively. The D’Ambrosio et al study reported 94% specificity and 100% sensitivity for the LoaScope. The Pedram et al study found that the Loa Ab test was 94% specific and 82–88% sensitive when read by eye, and 72% sensitive and 96–100% specific when read with a handheld reader using a cut-off of 600 reader units. However, the performance of the device can be improved by changing the cut-off. The data we analysed here used cutoff of 157 reader units, which has been shown to deliver specificity of >95% for other filarial infections [9].

### LoaScope and antibody *Loa loa* data in Gabon

**Fig 1:**
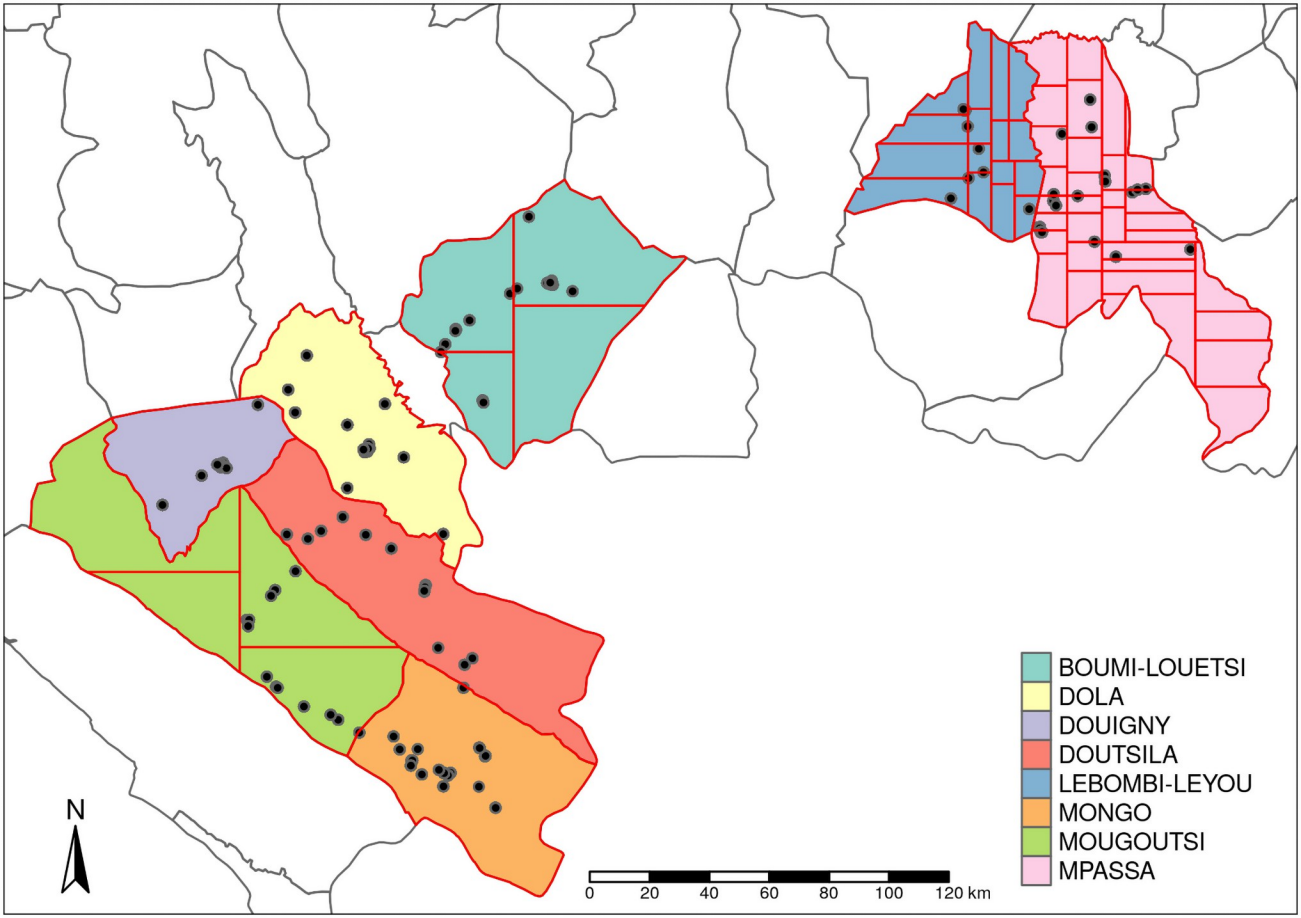
Gabon geography. Map showing the department (color shadings), evaluation units (EUs, red lines) and the locations of the 146 villages (black dots) surveyed in the southern part of Gabon. The Gabon shapefile was obtained from World Bank Data Catalog (https://data.humdata.org/dataset/geoboundaries-admin-boundaries-for-gabon).

The data used for this study are from *Loa loa* surveys conducted in Gabon between December 2017 and December 2018 [9]. Surveys were conducted in 146 villages over 8 departments (Fig 1), covering a total of 7,761 individuals aged 10 years or more. Empirical prevalences for these 146 villages have been reported in Ella *et al*. [9]. Data were obtained using both the Loascope and the Ab test, resulting in the following outcomes for each individual:

1. *Ab test*—a binary outcome representing the presence or absence of detectable antibodies to the Loa loa parasite;
2. *LoaScope measurement*—a binary/continuous outcome representing the presence or absence of microfilariae in a blood sample and, if present, the intensity of infection, expressed as the estimated number of microfilariae per millilitre of blood (mf).

For the analysis, we converted any LoaScope measurement less than 150 mf/ml to zero, because positive values less than 150 were considered unreliable and unlikely to be of clinical significance.

### Defining an evaluation unit

An evaluation unit (EU) needs to be large enough to be operationally practical but not so large that within-EU heterogeneity makes it highly unlikely that any EU can reliably be classified as safe. For these analysis, we decided to create EU’s whose total population size is between 5,000 and 15,000. As the smallest digitised boundary available to us was the department (admin 2) level, we used population density estimates at 100m resolution from WorldPop (https://www.worldpop.org/) to create a partition of each department into compact EUs of the required size (Fig 1).

### Exploratory analysis

**Fig 2:**
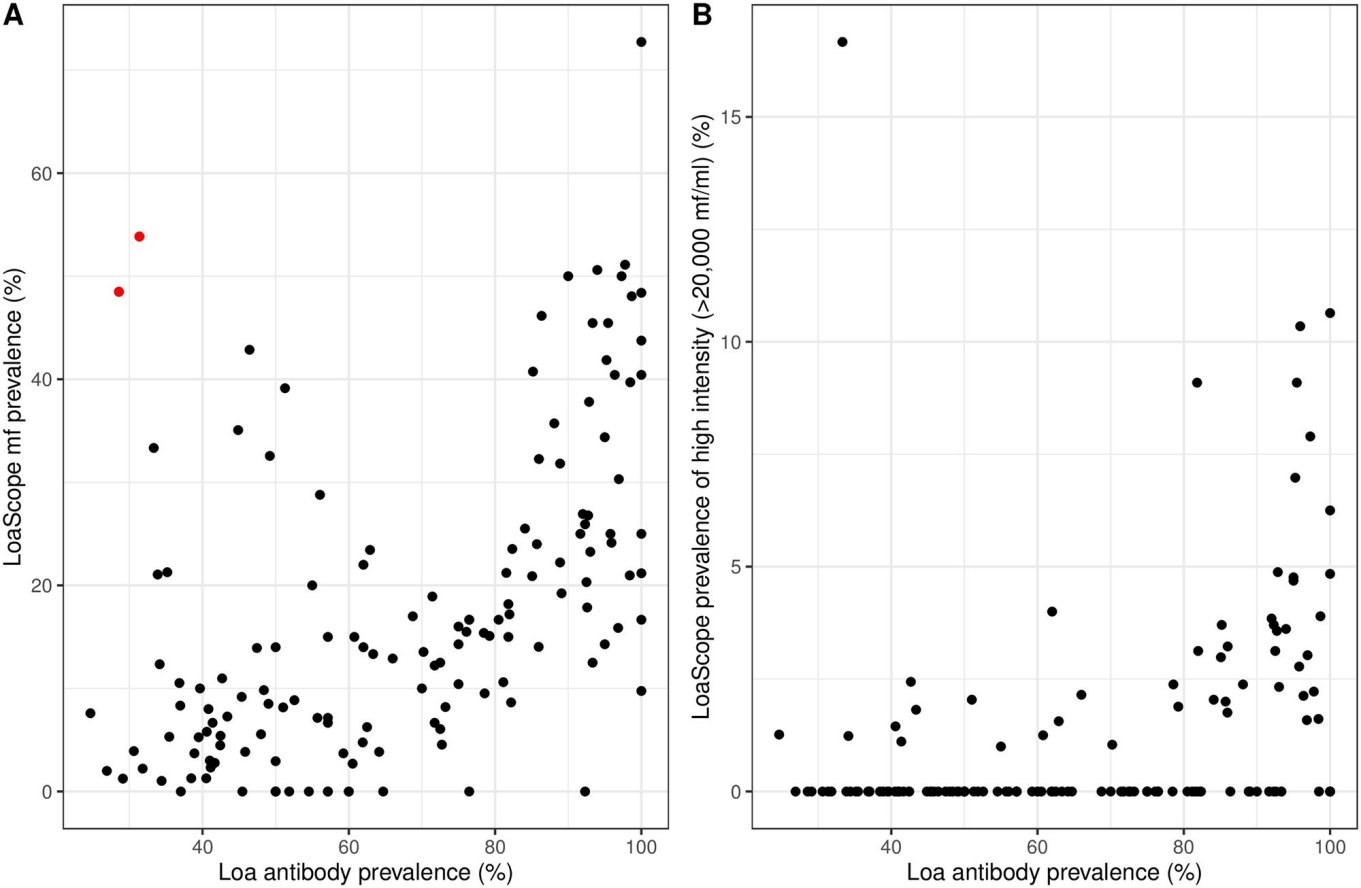
Empirical prevalence. Village-level empirical prevalence of Loa Ab plotted against LoaScope mf prevalence and the LoaScope prevalence of high intensity (> 20, 000 mf/ml).

An exploratory analysis was performed to establish that Loa Ab prevalence was correlated with LoaScope MF prevalence and with LoaScope high intensity (> 20, 000 mf/ml) prevalence. Village-level empirical prevalence was calculated as the ratio of the number of positive test results to the total number of people tested. Fig 2 shows the Village-level empirical prevalence of Loa Ab plotted against LoaScope mf prevalence and the LoaScope prevalence of high intensity. In both cases there is a positive association, which we now investigate in more detail by developing a joint geostatistical model.

Fig 2A reveals that in nearly every village, the prevalence of antibodies exceeds the mf prevalence. This is expected, because every infective larva (L3 stage, acquired upon fly bite) can trigger a serological response, without necessarily turning into a gravid female. The two villages highlighted in red are outliers falling in the opposite scenario, with the prevalence of antibodies (approximatively 30%) being well below the mf prevalence of mf (48–55% range). Given the high sensitivity of the Loa Antibody Rapid Test ([8], these 2 points do not appear to be plausible and may arise from incorrect data collection or entry. However, as we cannot verify that these two data-points are incorrect we retained them in the geostatistical analysis presented below.

### Geostatistical modelling framework

For the *j*^*th*^ sampled individual at location *x*_*i*_: *i* = 1, …, *n*, we denote by *Y*_1*j*_(*x*_*i*_) = 1 or 0 the binary Ab test outcome corresponding to a positive or negative result, respectively; by *Y*_2*j*_(*x*_*i*_) the LoaScope-derived estimate of the number of microfilariae per ml in a blood sample; and by *Y*_3*j*_(*x*_*i*_) the binary outcome with value 1 if *Y*_2*j*_(*x*_*i*_) ≥ 150 and 0 otherwise. As a convenient shorthand, we denote the complete sets of Ab, LoaScope intensity and LoaScope binary outcomes by *Y*_1_, *Y*_2_ and *Y*_3_, respectively, and write *Y* = (*Y*_1_, *Y*_2_, *Y*_3_).

The statistical model for a single binary outcome is necessarily a Bernoulli random variable; we write Prob(*Y*_1_ = 1) = *ρ* and Prob(*Y*_3_ = 1) = *π*. Our model for a single value of *Y*_2_ conditional on *Y*_3_ = 1 is a continuous probability distribution, *G*(*y*; λ, *κ*) = Pr(*Y*_2_ ≤ *y*|*Y*_3_ = 1). Following Giorgi *et al* [6] and Schluter *et al* [5] we assume that *G*(*y*; λ, *κ*) is the cumulative distribution function of a Weibull random variable with scale parameter λ and shape parameter *γ*.

To capture geographical variation in the disease process we allow each of the parameters *ρ*, λ and *π* to vary according to measured location-specific covariates (fixed effects) and unexplained residual spatial variation (random effects). Specifically,

$$\begin{array}{c} \text{log}\left[\rho_{i}/\left\{1-\rho_{i}\right\}\right]=\mu_{1}\left(x_{i}\right)+S_{0}\left(x_{i}\right), \end{array}$$ (1)

$$\begin{array}{c} \text{log}\left[\lambda_{i}\right]=\mu_{2}\left(x_{i}\right)+S_{1}\left(x_{i}\right)+\alpha_{1}S_{0}\left(x_{i}\right), \end{array}$$ (2)

$$\begin{array}{c} \text{log}\left[\pi_{i}/\left\{1-\pi_{i}\right\}\right]=\mu_{3}\left(x_{i}\right)+S_{2}\left(x_{i}\right)+\alpha_{2}S_{0}\left(x_{i}\right). \end{array}$$ (3)

In the above equations, the mean functions *μ*_*k*_(*x*) are linear regressions,

$$\mu_{k}\left(x_{i}\right)=\beta_{k0}+\beta_{k1}\text{EVI}\left(x_{i}\right)+\beta_{k2}\text{Elev}\left(x_{i}\right),k=1,2,3,$$

where EVI(*x*_*i*_) is the enhanced vegetation index (EVI) at location *x*_*i*_, Elev(*x*_*i*_) is the elevation (in metres) at location *x*_*i*_ and the *β* are regression parameters (fixed effects). Also, *S*_0_(*x*), *S*_1_(*x*) and *S*_2_(*x*) are zero-mean stationary Gaussian processes (random effects). Finally, *α*_1_ and *α*_2_ are scaling parameters. In addition to elevation and enhanced vegetation index (EVI), we considered the following other environmental covariates: normalized difference vegetation index (NDVI); rainfall; soil PH; night light emission; distance from closest water body; day and night land surface temperature. However, none of these led to any improvement in performance. This result was also found by Schlüter *et al* [5].

Two desirable features of this overall model structure are that it recognises the influence of environmental variables on local prevalence and, through the parameters *α*_1_ and *α*_2_, does not pre-suppose the strength of the cross-correlations among Ab presence/absence, LoaScope presence/absence and LoaScope intensity.

### Likelihood-based inference: Parameter estimation and prediction

We estimate the model parameters and their standard errors using Monte Carlo maximum likelihood (MCML) [10]. Maximum likelihood is known to be a statistically efficient method of parameter estimation. Monte Carlo maximum likelihood is a computationally intensive way of implementing maximum likelihood when the likelihood function is mathematically intractable. The likelihood function and the MCML procedure is presented in the supplementary material(S1 Appendix).

#### Prediction using LoaScope and Ab data

The predictive distribution of any unobserved quantity *T* is its conditional distribution given all of the observed data, *y*. We call *T* a *predictive target*.

Our eventual predictive target is the safety status of any designated area within the geographical region of interest, *R*. To achieve this, we first cover *R* with a set of square pixels that are small enough to capture all material variation in local prevalence, and denote by $X=\{x_{1}^{*},\ldots ,x_{q}^{*}\}$ the grid of pixel centres. For any grid-point x* we write *I*(x*) for the probability that an individual at location x* has intensity of infection greater than *c* = 20, 000 Mf/ml, computed as

$$\begin{array}{c} I\left(x^{*}\right)=\text{Pr}\left(Y_{2}\left(x^{*}\right)>c|W\right)=\pi \left(x^{*}\right)\text{exp}\left[-{\left\{c/\lambda \left(x^{*}\right)\right\}}^{\gamma }\right], \end{array}$$ (4)

where *W* is multivariate Gaussian with mean zero and covariance matrix as defined in Eq (1) in the supplementary material(S1 Appendix). The predictive target for any designated area *A* within *R* is the proportion of the population of *A* who are infected with at least 20,000 Mf/ml. This is

$$\begin{array}{c} T=\int_{A}m\left(x\right)I\left(x\right)\;dx, \end{array}$$ (5)

where *m*(*x*) is the population density at location *x*. An area is declared *safe* if Pr(*T* ≤ 0.01) > 0.95, *unsafe*, if Pr(*T* ≤ 0.01) < 0.05; and *don’t know* if 0.05 ≤ Pr(*T* ≤ 0.01) ≤ 0.95. In practice, we approximate *T* by quadrature over the set *X* of grid-points.

Predictive inference for *T* requires us to sample from the joint predictive distribution of *W*, and hence of *ρ*, λ and *π*, over the grid-points of *X*; we denote this by W* to distinguish it from the values of *W* at the *n* data-locations. To sample from the predictive distribution of W* we use a Metropolis-adjusted Langevin MCMC algorithm, to sample from the predictive distribution of *W*, then sample directly from the conditional distribution of W* given *W*. Sampling from the predictive distribution of *T* follows by direct substitution into Eqs (4) and (5).

In this paper we have used plug-in prediction, replacing the unknown parameters, *θ*, by their Monte Carlo maximum likelihood estimates. In principle, we should allow for parameter uncertainty either by weighting plug-in predictions for different values of *θ* by their approximate multivariate Normal sampling distribution or, if a suitable joint prior for *θ* is available, by using Bayesian inference. In practice, we have found that this makes little difference in the current context. Because prediction is driven primarily by local information, and parameter estimation by global information, prediction uncertainty dominates parameter uncertainty.

#### Prediction using only Ab test outcomes

We now consider the prediction of *T* using only the Ab test data alone. The first step is to fit the model in Eq (1) to the available Ab data, *Y*_1_. We then use the resulting parameter estimates and previously obtained estimates of the remaining parameters of the joint model to sample from the joint predictive distribution of W* conditional on *y*_1_,

$$\begin{array}{c} f\left(w^{*}|y_{1}\right)=\int_{R}f\left(w_{1}|y_{1}\right)f\left(w_{1}^{*}|w_{1}\right)f\left(w_{2}^{*}|w_{1}^{*}\right),f\left(w_{3}^{*}|w_{1}^{*}\right)\;dw_{1}. \end{array}$$

To achieve this, we first sample from *f*(*w*_1_|*y*_1_) using a Metropolis-adjusted Langevin MCMC algorithm, then sample directly from the multivariate Gaussian conditional distributions $f(w_{1}^{*}|w_{1})$, $f(w_{2}^{*}|w_{1}^{*})$ and $f(w_{2}^{*}|w_{1}^{*})$ and substitute the sampled values into Eqs (4) and (5).

#### Prediction using the two-stage strategy

Finally, we consider prediction using the two-stage strategy. The first step is to perform prediction using the Ab data alone and classify the EUs as *safe*, *unsafe* or *don’t know* for MDA. Then, use Ab data plus Loascope data to predict safety status of each EU classified as *don’t know*.

## Results

### Gabon data

In our analysis of the Gabon data we included enhanced vegetation index and elevation as covariates, as both were significantly associated with the Ab and LoaScope prevalence. Table 1 shows the fitted parameter estimates and 95% confidence intervals. The correlation between the Gaussian process *S*_0_ common to the three outcomes and the processes *S*_1_ and *S*_2_ that relate to LoaScope intensity and presence/absence are 0.5(0.2–0.8) and 0.7(0.6–0.9), respectively.

**Table 1:** Monte Carlo maximum likelihood estimates and corresponding 95% confidence intervals resulting from the joint modelling as described in section geostatistical modelling framework.

Parameter	Estimate	95% CI
*β*_10_ (Intercept)	0.817	(0.760, 0.875)
*β*_11_ (EVI)	0.494	(0.426, 0.562)
*β*_12_ (Elevation)	0.232	(0.163, 0.300)
*β*_20_ (Intercept)	7.964	(7.862, 8.066)
*β*_21_ (EVI)	0.148	(0.057, 0.240)
*β*_22_ (Elevation)	0.032	(-0.065, 0.130)
*β*_30_ (Intercept)	-1.656	(-1.729, -1.584)
*β*_31_ (EVI)	0.434	(0.363, 0.505)
*β*_32_ (Elevation)	0.074	(0.001, 0.148)
$\sigma_{0}^{2}$	0.516	(0.333, 0.700)
$\sigma_{1}^{2}$	0.547	(0.388, 0.706)
$\sigma_{2}^{2}$	0.766	(0.518, 1.014)
*ϕ*_0_	5.776	(2.897, 8.654)
*ϕ*_1_	1.468	(0.748, 2.188)
*ϕ*_2_	4.344	(2.353, 6.335)
*α*_1_	0.532	(0.246, 0.817)
*α*_2_	0.689	(0.568 0.940)
*γ*	0.661	(0.631 0.690)

**Fig 3:**
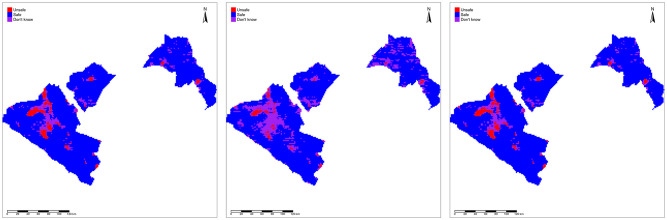
Pixel-level map. Map showing the classification as safe (blue), unsafe (red) or don’t know (purple) for MDA at the pixels using “LoaScope and Ab data” (left panel), “Ab data alone” (middle panel) and “Two-stage strategy” (right panel). The Gabon shapefile was obtained from World Bank Data Catalog (https://data.humdata.org/dataset/geoboundaries-admin-boundaries-for-gabon).

**Fig 4:**
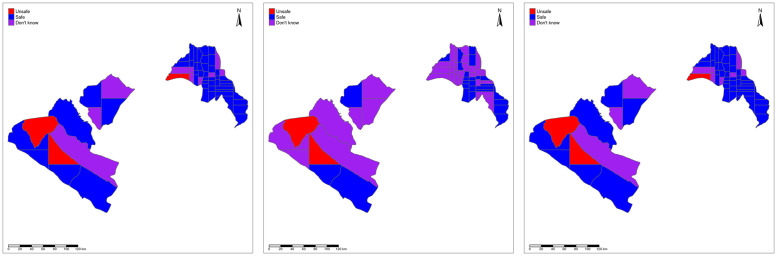
EU-level map. Map showing the classification as safe (blue), unsafe (red) or don’t know (purple) for MDA at the EUs using “LoaScope and Ab data” (left panel), “Ab data alone” (middle panel) and “Two-stage strategy” (right panel). The Gabon shapefile was obtained from World Bank Data Catalog (https://data.humdata.org/dataset/geoboundaries-admin-boundaries-for-gabon).

**Fig 5:**
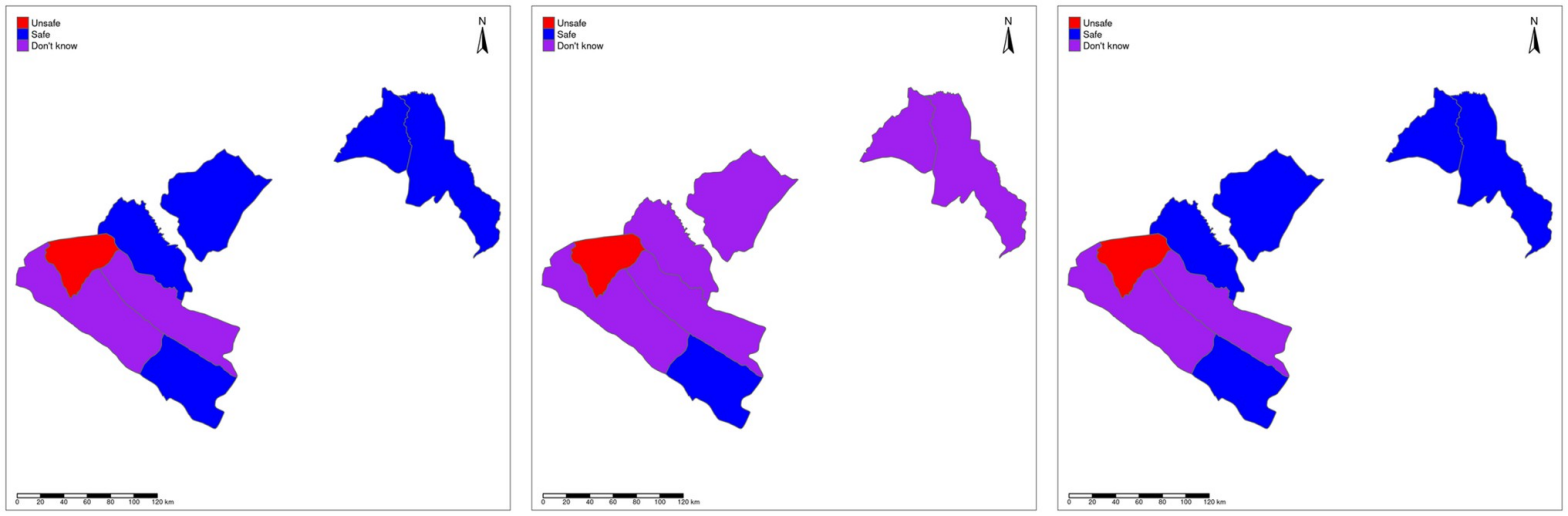
Department-level map. Map showing the classification as safe (blue), unsafe (red) or don’t know (purple) for MDA at the departments using “LoaScope and Ab data” (left panel), “Ab data alone” (middle panel) and “Two-stage strategy” (right panel). The Gabon shapefile was obtained from World Bank Data Catalog (https://data.humdata.org/dataset/geoboundaries-admin-boundaries-for-gabon).

We consider prediction at three levels of spatial aggregation: on a regular 2km by 2km pixel grid covering all 8 departments; on the EU partitions; and on the department partitions. In Figs 3–5, we map the resulting safety classifications using: the LoaScope and Ab data; only Ab data; and the two-stage strategy at pixel- level (Fig 3), EU-level (Fig 4) and department level (Fig 5). The three pixel-level classifications are very similar. Unsurprisingly, the aggregated classifications show bigger discrepancies, as each crossing of the 0.95 probability threshold affects the classification of a larger area. However, at the department level the two-stage strategy and using both LoaScope and Ab data give similar classifications and imply that only Ab testing would be required in four departments (Doutsila, Douigny, Mougoutsi and Mongo) out of the eight considered.

Table 2 shows the numbers of pixels that our model-based predictions classify as *safe*, *unsafe* and *don’t know* for MDA using LoaScope and Ab data or Ab data alone (left panel) and using LoaScope and Ab data or the two-stage strategy (right panel). Table 3, shows the same information at EU-level. The concordance between using both LoaScope and Ab data from all villages and the more economical two-stage strategy is perfect at EU-level and near-perfect at pixel-level (11 discordances out of 19,640 pixels).

**Table 2:** Contingency table comparing the performance of the three strategies at the pixel-level. *LS and Ab* denotes using the joint model of LoaScope and Ab data; *Ab only* denotes using the Ab data only, *two-stage* denotes using the Ab data first, followed by joint analysis of LoaScope and Ab data to re-classify the *don’t know* (DK) areas.

		**Ab only**					**Two-stage**		
		Unsafe	Safe	DK			Unsafe	Safe	DK
**LS and Ab**	Unsafe	580	0	432	**LS and Ab**	Unsafe	1012	0	0
	Safe	0	14629	2042		Safe	0	16671	0
	DK	6	5	1946		DK	6	5	1946

**Table 3:** Contingency table comparing the performance of the three strategies at the EU-level. *LS and Ab* denotes using the joint model of LoaScope and Ab data; *Ab only* denotes using the Ab data only, *two-stage* denotes using the Ab data first, followed by joint analysis of LoaScope and Ab data to re-classify the *don’t know* (DK) areas.

		**Ab only**					**Two-stage**		
		Unsafe	Safe	DK			Unsafe	Safe	DK
**LS and Ab**	Unsafe	2	0	1	**LS and Ab**	Unsafe	3	0	0
	Safe	0	26	21		Safe	0	47	0
	DK	0	0	10		DK	0	0	10

The results in Table 3 suggest that, in the context of Gabon, the two-stage strategy results in identical safety classifications as the joint LoaScope and Ab while saving significant resources. In particular, the Table 3 results for the Ab only classifications show that 28 EUs could be classified as unsafe (n = 2) and safe (n = 26) from Ab data alone, while 32 were classified as don’t know. For these 32 uncertain EUs, adding LoaScope in the second stage resulted in 1 additional EU classified as unsafe and an additional 21 EUs classified as safe. Taken together, this suggests that the two-stage strategy avoided the need for LoaScope in 47% of the EUs (28 out of 60) while resulting in identical safety classifications. When safety classification is made at a smaller scale (pixel-level results in Table 2), the data suggest that the two-stage strategy had 99.9% agreement (19629/19640 pixels) compared to the joint LoaScope and Ab strategy. Where there is discordance, the two-stage strategy classified 6 pixels as unsafe and 5 pixels as safe that the joint strategy would have classified as don’t know; importantly, no pixels classified unsafe by the joint strategy were classified as safe, suggesting that even if classification predictions were extended to the village level (approximated by the 2km by 2km pixels), safe treatment decisions would be maintained.

### Simulation study

Because the data used here are indicative of just one highly endemic loa loa setting, we conducted a simulation study to understand how these diagnostic strategies would perform in other endemic settings. Our simulation study had the following two aims: to investigate how well we can delineate an area as *safe* or *unsafe* using both LoaScope and Ab data or only Ab data; and to evaluate different sampling strategies that can achieve an acceptable level of uncertainty around the decision to implement MDA.

We simulated data using the model fitted to the Gabon data, creating different sampling scenarios by varying the number of villages, the number of people sampled in each village and, through adjustments to the regression intercepts in Eqs (1), (2) and (3), the percentage of EUs that are *safe* and *unsafe* according to the actual simulated prevalence and intensity surfaces.

The sampling strategies considered were:

- *Number of villages surveyed per department*: We considered 100% of the surveyed villages across the entire geographic area (n = 146), 12 sites per department (n = 96) and 6 sites per department (n = 48).
- *Number of individuals surveyed per village*: We considered sampling 30, 50 and 70 number of people per village.
- *Percentage of safe EUs*: Using the estimates from the fitted model in the 60 EUs, we simulated different ‘true’ prevalence surfaces by varying the percentage of EUs that are safe for MDA. We considered scenarios when 25%, 50% and 85% of the EUs are safe and when 8%, 25% and 50% of the EUs are unsafe. This is done in order to understand how the sampling will perform in different settings.

We evaluated the performance of the different scenarios using the proportion of EUs that are classified correctly as safe and unsafe. Specifically, this is defined as the number of EUs classified as safe by our model divided by the total number of EUs (which is 60 in this case). An EU is declared as safe if the probability that at most 1% of the population in the EU who are infected with at least 20,000 Mf/ml is greater than 95%.

**Fig 6:**
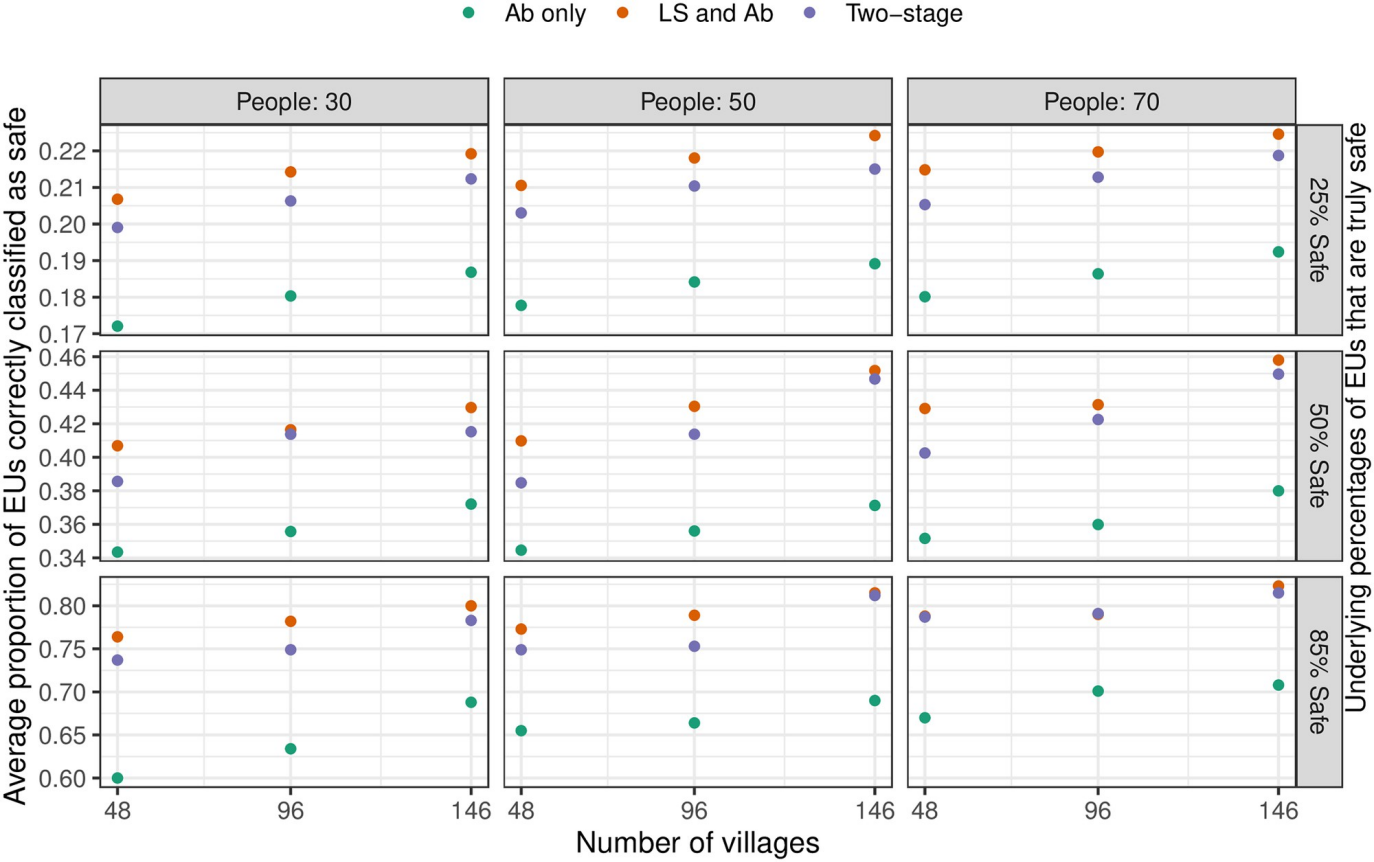
Simulation result for safety classification. Plot showing the proportion of correctly classified EUs as safe for different combinations of the number of sampled villages and the number of people sampled per village, using both LoaScope and Ab data (orange dots), only Ab data (green dots) or two-stage strategy (purple dots). Note that 146 is the total number of surveyed villages across the entire geographic area; 96 corresponds to 12 villages per department; and 48 corresponds to 6 villages per department.

**Fig 7:**
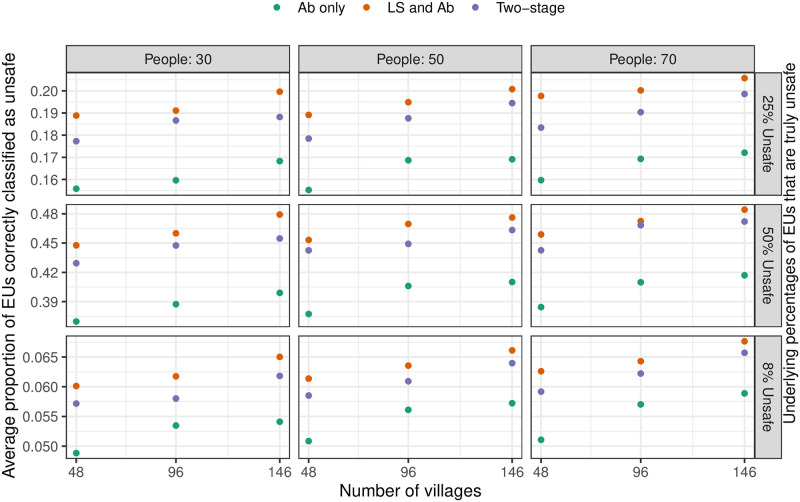
Simulation result for unsafety classification. Plot showing the proportion of correctly classified EUs as unsafe for different combinations of the number of sampled villages and the number of people sampled per village, using both LoaScope and Ab data (orange dots), only Ab data (green dots) or two-stage strategy (purple dots). Note that 146 is the total number of surveyed villages across the entire geographic area; 96 corresponds to 12 villages per department; and 48 corresponds to 6 villages per department.

The results of the simulation are summarised in Figs 6 and 7. Increasing the number of villages sampled is more beneficial than increasing the number of individuals sampled in each village. The two-stage strategy delivers only slightly lower proportions of correctly classifieds pixels than does the LS and Ab strategy, whereas the Ab alone strategy results in substantially lower proportions of correct classifications. There is a little discernible difference in performance across the 3 safety or unsafety levels and this suggests that the performance of the strategies is more dependent on geographical variation and less dependent on underlying prevalence.

## Discussion

We have developed a novel multivariate geostatistical model to analyse data from two diagnostic tools (LoaScope and Ab test) with the aim of delineating whether an area is safe for MDA or not. We have shown that using this model when only Ab data are available can deliver reasonably accurate assessments of the safety of an area for MDA by exploiting both the spatial correlation between locations [6] and the statistical association between Ab and Loascope outcomes. However, our results more strongly support the use of a two-stage strategy, in which Ab testing is used to identify areas that, with acceptably high probability, are safe or unsafe for MDA, followed by Loascope testing in areas whose safety status is unclear. For given numbers of sampled villages and individuals, this strategy appears almost to match the performance of a more expensive strategy that requires data on both Ab and LoaScope testing to be collected at every sampled location, and may therefore represent a more cost-effective use of limited resources for field data-collection. This work therefore contributes to the global effort towards the elimination of onchocerciasis as a public health problem by potentially reducing the time and cost required to establish whether an area is safe for MDA.

A limitation of our findings is that they apply to the particular structure of the geographical variation in *Loa loa* prevalence and intensity that we observed from the Gabon data. Nevertheless, the results of the present paper constitute a prima facie case for further investigation of a two-stage testing strategy based on a statistically efficient and cost-effective combination of LoaScope and Ab diagnostic tools. To this end, we are currently evaluating the robustness of the Gabon model by applying it to data from other countries and will report the results in due course.

A second limitation is that the predictions presented in this paper do not account for migration. There will always be a chance that individuals with high intensity Loa loa infections could move into an area predicted to be of low risk and thus have the potential to receive MDA. To mitigate this potential risk, we recommend that drug distributors ask individuals their length of residency and test, using either LoaScope or microscopy, anyone found to have previously resided in an area of greater Loa loa risk prior to receipt of MDA.

For the present study we created a set of compact EUs that do not correspond to any administrative boundaries or intervention units. We suggest that future users of our proposed two-stage approach may want to consider sub-district (admin3) administrative boundaries as EUs to better operationalize both testing and treatment decisions at the local level. However, an important benefit of the framework presented here is that it is agnostic to EU formation. In areas where the prevalence of Loa loa is expected to be low, programs may opt to use larger EUs for prediction (e.g. district or region) to reduce the initial sampling burden.

A current obstacle to achieving full geographic scale up of ivermectin treatment to areas where onchocerciasis is endemic is the lack of a safe and efficient strategy for mapping loaiasis. The recent advent of the LoaScope has led to a TaNT strategy for safe individual treatment where high intensity loaiasis infections are common; however, such a strategy would be impractical to implement across the geographical expanse where Loa loa is possibly endemic. In this paper we demonstrate how the introduction of a new loa antibody rapid test, coupled with geostatistical modeling, can lead to more resource efficient, yet equally safe, treatment decisions at varying geographic scales. In particular, the results presented here suggest that a two-stage strategy, whereby the rapid antibody tool is used to test a sample of adults from a few villages per sub-district and only when the resulting safety classification is uncertain is more resource-intensive LoaScope testing required, performs similarly well to when both diagnostics are applied at the onset. This represents an important advancement in developing a feasible, safe and efficient strategy for mapping loaiasis and, subsequently, a significant contribution to the global effort towards the elimination of onchocerciasis as a public health problem.

## Supporting information

S1 Appendix Geostatistical modelling equations. (PDF)
