# Supplementary material for: Geostatistical modelling enables efficient safety assessment for mass drug administration with ivermectin in Loa loa endemic areas through a combined antibody and LoaScope testing strategy for elimination of onchocerciasis
Source: PLoS Negl Trop Dis. 2022 Feb 9;16(2):e0010189. doi: 10.1371/journal.pntd.0010189 (PMC8863288; doi:10.1371/journal.pntd.0010189)
Supplement: S1 Appendix — (PDF) [file pntd.0010189.s001.pdf]

S1 Appendix

A Geostatistical modelling equations

A.0.1 The multivariate distribution of the random effects

Let $W_{1i} = S_0(x_i)$, $W_{2i} = \alpha_1 S_0(x_i) + S_1(x_i)$ and $W_{3i} = \alpha_2 S_0(x_i) + S_2(x_i)$ as defined in Eq (1), (2) and (3) in the main manuscript. For each of $k = 0, 1, 2$ write $W_k = \{W_{ki} : i = 1, ..., n\}$, and $W = (W_1, W_2, W_3)$ The distribution of W is multivariate Gaussian with mean zero and covariance matrix

$$\Sigma = \begin{pmatrix} \Sigma_0 & \alpha_1\Sigma_0 & \alpha_2\Sigma_0 \\ \alpha_1\Sigma_0 & \Sigma_1 + \alpha_1^2\Sigma_0 & \alpha_1\alpha_2\Sigma_0 \\ \alpha_2\Sigma_0 & \alpha_1\alpha_2\Sigma_0 & \Sigma_2 + \alpha_2^2\Sigma_0 \end{pmatrix}. \quad (1)$$

Each sub-matrix Σ_k is of form $\sigma_k^2 R(\phi_k) : k = 0, 1, 2$ where σ_k^2 is the variance of the Gaussian process $S_k(x)$, $R(\phi)$ is a correlation matrix with elements $r_{ij} = \exp(-u_{ij}/\phi)$ and u_{ij} is the Euclidean distance between locations x_i and x_j.

The likelihood function and parameter estimation

Let $f(y|w;\theta)$ denote the probability distribution of an observable set of random variables Y conditional on the value of an unobservable set of random effects W, indexed by a vector of parameters, θ. The likelihood function for θ, written $L(\theta)$, is the probability distribution of Y considered as a function of θ with y fixed at its observed value, hence

$$L(\theta) = \int f(y|w;\theta)g(w;\theta)\, dw$$

where $g(w;\theta)$ is the unconditional distribution of W.

For our model, Y denotes the complete set of Ab test presence/absence outcomes, LoaScope presence/absence outcomes and LoaScope measured intensities. Also, these three quantities are conditionally independent given W. Hence, the likelihood function is of the form

$$L(\theta) = \int f(y_1|w_1;\theta)f(y_2|w_2;\theta)f(y_3|w_3;\theta)g(w;\theta)dw. \quad (2)$$

The high-dimensional integral in Eq (2) cannot be solved analytically. We use Monte Carlo methods to approximate it as follows. We first let θ_0 denote our

best guess for the true value of θ, write the log-likelihood-ratio function as

$$\ell(\theta) = \log\{L(\theta)/L(\theta_0)\},$$

where $L(\theta)$ is defined at (2), and note that for any fixed value θ_0 the maximum likelihood estimator is the value of θ that maximises $\ell(\theta)$. Next, we generate N samples, $w^{(j)} = \{w_1^{(j)}, w_2^{(j)}, w_3^{(j)}\}, j = 1, ..., N$, from the conditional distribution of W given y and $\theta = \theta_0$ using a Metropolis-adjusted Langevin MCMC algorithm [1] and approximate $\ell(\theta)$ by the quantity

$$\ell_N(\theta) = \log\left\{\frac{1}{N}\sum_{j=1}^{N}\frac{f(y_1|w_1^{(j)};\theta)f(y_2|w_2^{(j)};\theta)f(y_3|w_3^{(j)};\theta)g(w^{(j)};\theta)}{f(y_1|w_1^{(j)};\theta_0)f(y_2|w_2^{(j)};\theta_0)f(y_3|w_3^{(j)};\theta_0)g(w^{(j)};\theta_0)}\right\}. \quad (3)$$

Numerical maximisation of $\ell_N(\theta)$ delivers the Monte Carlo maximum likelihood estimate, $\tilde{\theta}$. If our best guess θ_0 happens to be equal to the maximum likelihood estimate $\hat{\theta}$, then $\tilde{\theta} = \hat{\theta}$ and $\ell_N(\tilde{\theta}) = 0$. The value of $\ell_N(\tilde{\theta})$ is therefore an indirect measure of how closely $\tilde{\theta}$ approximates $\hat{\theta}$. If necessary, we can replace θ_0 by $\tilde{\theta}$ and repeat the numerical maximisation of $\ell_N(\theta)$ with a larger value of N to obtain a closer approximation to $\hat{\theta}$.

References

[1] Gareth O Roberts, Andrew Gelman, Walter R Gilks, et al. Weak convergence and optimal scaling of random walk metropolis algorithms. *The annals of applied probability*, 7(1):110–120, 1997.
